# Astrocytes and Memory: Implications for the Treatment of Memory-related Disorders

**DOI:** 10.2174/1570159X22666240128102039

**Published:** 2024-01-29

**Authors:** Juan Wang, Ping Cheng, Yan Qu, Guoqi Zhu

**Affiliations:** 1 Key Laboratory of Xin’an Medicine, The Ministry of Education and Key Laboratory of Molecular Biology (Brain Diseases), Anhui University of Chinese Medicine, Hefei 230012, China

**Keywords:** Astrocyte, memory, memory-related disorder, synaptic plasticity, neuronal networks, Alzheimer’s disease, PTSD

## Abstract

Memory refers to the imprint accumulated in the brain by life experiences and represents the basis for humans to engage in advanced psychological activities such as thinking and imagination. Previously, research activities focused on memory have always targeted neurons. However, in addition to neurons, astrocytes are also involved in the encoding, consolidation, and extinction of memory. In particular, astrocytes are known to affect the recruitment and function of neurons at the level of local synapses and brain networks. Moreover, the involvement of astrocytes in memory and memory-related disorders, especially in Alzheimer’s disease (AD) and post-traumatic stress disorder (PTSD), has been investigated extensively. In this review, we describe the unique contributions of astrocytes to synaptic plasticity and neuronal networks and discuss the role of astrocytes in different types of memory processing. In addition, we also explore the roles of astrocytes in the pathogenesis of memory-related disorders, such as AD, brain aging, PTSD and addiction, thus suggesting that targeting astrocytes may represent a potential strategy to treat memory-related neurological diseases. In conclusion, this review emphasizes that thinking from the perspective of astrocytes will provide new ideas for the diagnosis and therapy of memory-related neurological disorders.

## 1 INTRODUCTION

**Fig. (1):**
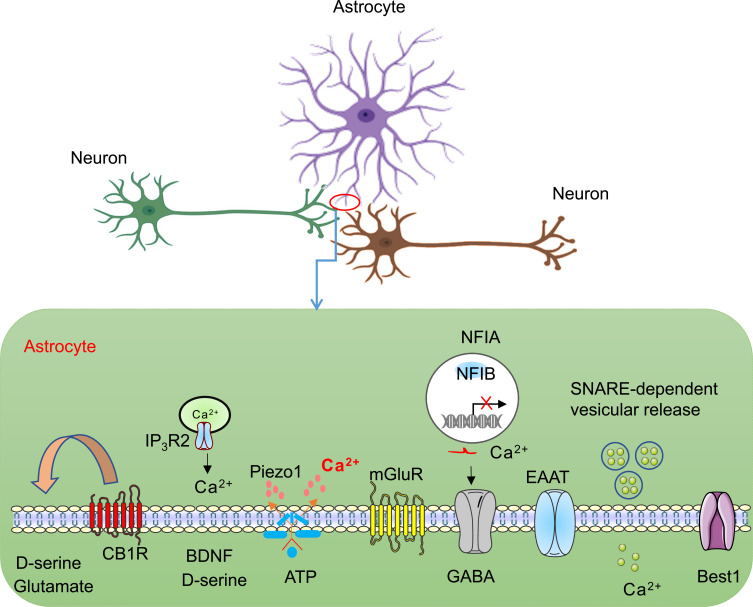
Mechanisms involved in the regulation of astrocytes in synaptic plasticity.

Over the last few decades, research activities in the field of memory have predominantly focused on neurons. However, the brain is a highly evolved organ and not just a collection of neurons and neural circuits. Consequently, it is necessary to consider the multiple types of constituent cells and their interactions, as these are crucial for the functionality of the entire system. Previously, astrocytes were not thought to actively participate in the processing of memory, possibly because they lack electrical excitability and have no direct connections with the surrounding sensory organs. However, since the 1990s, studies have shown that astrocytes, as an important aspect of the ‘tripartite synapse’, not only provide stable support for neurons but also sense synaptic activity, respond to surrounding synaptic activity through Ca^2+^ signals and then regulate neuronal activity *via* feedback and the release of neurotransmitters [1-4]. The astrocyte membrane features a variety of ion channels, transporters, ion pumps and receptors; collectively, these structures enable astrocytes to sense the activities of the surrounding neurons, regulate the balance of extracellular potassium ions, rapidly clear neurotransmitters in the synaptic gap, and provide metabolic support for neurons [5]. In addition, astrocytes can also release various neuroactive substances, including glutamate, ATP, γ-aminobutyric acid (GABA), and D-serine; these substances may play roles in regulating neuronal synaptic plasticity [6] (Fig. **1**).

Astrocytes are widely recognized to perform prominent roles in synaptogenesis and brain synaptic plasticity [7], and different steps or processes of memory [8, 9]. Moreover, astrocytes are known to participate in memory-related disorders, including Alzheimer’s disease (AD) [10, 11], post-traumatic stress disorder (PTSD) [12], and addiction [13]. With the development and application of various technologies, such as designer receptors exclusively activated by designer drug techniques [14], it is now possible to manipulate astrocytes in a highly dynamic manner. Furthermore, astrocytes can be monitored in real-time in the brain, thus providing opportunities for in-depth research targeting the role of astrocytes in different stages of memory [15, 16]. In this review, we demonstrate the unique contributions of astrocytes to synaptic plasticity and neuronal networks and discuss the role of astrocytes in various forms of memory processing. In addition, we also explore the pathogenesis and treatment of memory-related disorders, including AD, brain aging, addiction, and PTSD, from the perspective of astrocytes. This review highlights important implications for the treatment of memory-related neurological diseases.

## 2 ASTROCYTES AND CA^2+^ SIGNALING

Unlike neurons, astrocytes are mostly electrically silent cells. Their resting membrane potential rarely deviates from the K^+^ balance potential by more than a few millivolts, and there is no evidence that astrocytes exhibit propagating or gradient electrical signals that are similar to the electrical signals in neurons [17]. In the absence of obvious electrical signals, the excitability of astrocytes is mainly based on the highly spatiotemporal fluctuations of intracellular Ca^2+^ concentrations which are dependent on the cell membrane and intracellular channels [18]. Therefore, understanding intracellular Ca^2+^ signaling in astrocytes is crucial if we are to decode the functionality of astrocytes in the central nervous system (CNS).

With the development of advanced optical technologies such as two-photon excitation fluorescence imaging and sensitive gene coding indicators, Ca^2+^ signaling has become the focus for studying molecular and cellular aspects of astrocytes [19]. In the brain, Ca^2+^ events can occur spontaneously in the astrocytes [20-23]; these events can also be triggered by external physical stimuli [5, 23-25]. Astrocytes are known to express a large number of neurotransmitter receptors, many of which are G protein-coupled receptors (GPCR). Neurotransmitters can stimulate an increase in the intracellular concentration of Ca^2+^ in astrocytes; this occurs *via* the release of Ca^2+^ from the endoplasmic reticulum through the IP3 receptor [26-28]. In astrocytes, Ca^2+^ signaling transduction is dependent on type 2 IP3 receptor (IP_3_R2). However, even in the absence of IP_3_R2, astrocytes can still exhibit different types of Ca^2+^ fluctuations [29]. Ca^2+^ signals in astrocytes can be spatially confined to a single subcellular domain or generated in a coordinated manner by a number of astrocytes [30]. Super-resolution imaging has revealed many ring-like structures in the spongiform domain of astrocytes; these structures are referred to as ‘nodes’. These ‘nodes’ are morphological structures that carry Ca^2+^ transients on the tripartite synapse [31].

Ca^2+^ release in astrocytes occurs in a slow and spatially distributed manner. These events are thus considered to be too slow to participate in the processing of information in real-time. However, the application of advanced imaging and analysis techniques in recent studies has shown that the Ca^2+^ events that occur in parts of the microdomain of astrocytes are ultra-fast and last less than 300 ms; this is almost as fast as those of neurons (~208 ms) [32, 33]. In addition, astrocytes can also encode information by Ca^2+^ event patterns. Imaging data has shown that astrocytes possess a behavioral-dependent hot spot of Ca^2+^ activity, which remains stable for a few days and may represent a memory imprint [32]. In addition, the pattern of Ca^2+^ in astrocytes (that is, the total area, number, and duration of Ca^2+^ events) changes in each imaging frame [34]. Therefore, the spatial pattern of astrocyte activity can change almost instantaneously, thus implying that astrocytes may play an important role in the processing of information [19].

## 3 ASTROCYTES CAN INFLUENCE SYNAPTIC PLASTICITY

Synaptic connections can undergo several types of alterations, including formation, elimination, enhancement, and weakening [35, 36]. For example, the growth of new synaptic connections and the recombination of existing synaptic connections are considered key mechanisms for acquiring and stabilizing new memories [37]. Synaptic plasticity is used to describe changes in the strength of synaptic connections in the process of experiencing events and neural activities [38]. Such changes in the strength of synaptic connections are not only regulated by bidirectional communication between presynaptic and postsynaptic neurons, but also by interactions between neurons and their associated glia [39]. Astrocytes are important regulators of synaptic plasticity. Here, we discuss the important contribution of astrocytes to synaptic plasticity (Fig. **1**).

### 3.1 Long-term Potentiation (LTP)

LTP refers to the long-term enhancement of synaptic efficacy caused by high-frequency stimuli (HFS) [40]. LTP can be further divided into early LTP (E-LTP) and late LTP (L-LTP). E-LTP is usually induced by a single HFS, while L-LTP is induced by repeated HFS at second intervals [41, 42]. Decoding the Ca^2+^ signals produced by astrocytes is the key to understanding the role of these cells in LTP. The IP_3_R2 is considered to be the main receptor regulating Ca^2+^ signal transduction in astrocytes. LTP and glial transmission depend on Ca^2+^ release by IP_3_Rs in astrocytes [43]. In a previous study, Navarrete *et al.* found that Ca^2+^ signaling in astrocytes is necessary for choline-induced synaptic plasticity. These authors also found that choline-induced LTP required Ca^2+^ levels to increase in astrocytes, thus stimulating astrocytes to release glutamate and activate metabotropic glutamate receptors (mGluRs). When astrocyte Ca^2+^ signals were blocked, choline-induced LTP was not detected in IP_3_R2 knockout (KO) mice and wild-type mice [44]. In addition, Liu *et al.* used astrocyte IP_3_R2 KO mice, Mas-related gene A1 (MrgA1^+^) mice, and IP_3_R2 conditional KO mice to further investigate the role of astrocyte Ca^2+^ signals in synaptic plasticity. These authors showed that astrocyte IP_3_R2-dependent Ca^2+^ signals were critical for L-LTP rather than E-LTP. This process is mediated by brain-derived neurotrophic factor (BDNF) released by the astrocytes. Moreover, inhibiting IP_3_R2 signals or BDNF production in astrocytes damaged remote memory, while the deletion of BDNF in neurons led to defects in memory acquisition and retrieval [45]. In another study, Requeie *et al.* found that astrocytes mediated a new form of synaptic plasticity in the dopamine neurons within the ventral tegmental area (VTA) [46]. These authors also found that the burst discharge of a single dopamine neuron induced LTP in the excitatory synapses of adjacent dopamine neurons in the VTA in a manner that depended on the IP_3_R2-mediated increase of Ca^2+^ in astrocytes. Further research found that LTP needed to be co-located in the same astrocyte mediated by endogenous cannabinoid CB_1_ and dopamine D2 receptors and required the activation of presynaptic metabotropic glutamate receptor (mGluR1). The activation of astrocytes through chemical genetics has been shown to increase the burst discharge of DA neurons in the VTA [46].

Astrocytes mediate LTP by releasing fast mediators that change synaptic function. For example, the induction of NMDA receptor (NMDAR)-dependent LTP in hippocampal CA1 excitatory synapses requires the transient release of D-serine from local astrocytes [47]. D-serine is a physiological co-agonist of NMDAR and plays an important role in synaptic plasticity, learning, and memory [48, 49]. The mechanisms responsible for the direct release of D-serine from astrocytes have yet to be determined. Initially, it was thought that astrocytes released L-serine and L-serine, which were then shuttled to neurons, thus providing fuel for neurons to synthesize D-serine [50]. However, it cannot be denied that the induction of LTP in the hippocampus requires Ca^2+^ signals in astrocytes and D-serines [43, 47, 51]. The authors of a recent study claimed that astrocytes might act as the target cells for the activity-dependent production of D-serine and that the activation of astrocytic rather than neuronal α7-nicotine acetylcholine receptor (α7nAChR) promotes the occupation of binding sites for the D-serine-mediated activation of NMDAR [51]. In addition, astrocytes can also synthesize D-serine and release it through the Best1 calcium channel [52]. In another study, Huang *et al.* identified a transcription factor, nuclear factor I-A (NFIA), that could bind to DNA and regulate gene expression. The deletion of NFIA selectively reduced the morphological complexity and Ca^2+^ activity of hippocampal astrocytes. Furthermore, Theta burst stimulation (TBS)-induced LTP in the hippocampal Schaffer collaterals pathway was significantly inhibited in NFIA conditional KO mice; with the reduction of Ca^2+^-dependent D-serine, these mice showed impairment of working memory. In contrast, this impairment of LTP could be repaired by restoring astrocyte Ca^2+^ or by administering exogenous D-serine [53].

L-lactic acid is also known to be released by astrocytes and has been proven to play a key role in hippocampal LTP [54]. In the brain, metabolic coupling is considered a key mechanism for the active interaction between astrocytes and neurons in order to respond to neuronal activity. Glycogen stored in astrocytes is metabolized to L-lactic acid, which is then shuttled to neurons when energy is required [55-57]. Suzuki *et al.* found that the pharmacological inhibition of glycogen breakdown during LTP induction can disrupt the maintenance of hippocampal LTP, while the supplementation of L-lactate can rescue this damage [54]. Therefore, the availability of L-lactic acid is necessary for maintaining LTP.

Astrocytes can also affect LTP *via* the cannabinoid receptor type 1 (CB_1_R) signaling pathway. CB_1_R has been identified as a specific type of cannabinoid receptor and is expressed at high levels in neurons throughout the entire brain [58]. CB_1_R is also functionally expressed in astrocytes. The activation of CB_1_R stimulates the release of Ca^2+^-dependent glutamate and enhances synaptic transmission at the hippocampal CA3-CA1 synapse [59]. The supply of D-serine mediated by astrocyte CB_1_ receptor is a necessary condition for hippocampal LTP [60]. Mutant mice lacking astrocytic CB_1_R (GFAP-CB_1_-KO) generated an abnormal form of hippocampal LTP in the CA3-CA1 synapse that could be rescued by the supplementation of exogenous D-serine [60]. Astrocytes express a variety of receptors that transmit signals through the second messenger, cAMP. Zhou *et al.* developed a method that used light to activate adenylate cyclase to promote the levels of cAMP in astrocytes *in vivo*. These authors found that increasing the levels of cAMP in hippocampal astrocytes at different time points was sufficient to induce LTP in peripheral neurons and promote the formation of memory and that this effect could be interrupted by blocking NMDAR-dependent synaptic plasticity [61].

Very recent research demonstrated that astrocytes express Piezo1, a mechanically gated cation channel that can mechanically regulate Ca^2+^ signals and ATP release in astrocytes. The knockout of Piezo1 in astrocytes led to the impairment of hippocampal LTP and neurogenesis. In addition, Piezo1-deficient mice showed impairments of spatial working memory. In contrast, the overexpression of Piezo1 in astrocytes was shown to rescue the damaged LTP and memory impairment [62]. Similarly, the occurrence of LTP can also affect the state of astrocytes. For example, Henneberger *et al.* found that high-frequency stimulation (HFS) of the Schafer collateral of the hippocampus induced a reduced volume in the perisynaptic astroglial processes (PAPs), promoted the withdrawal of PAPs on the nanoscale, thereby promoting an overflow of extracellular glutamate and enhancing the activation of NMDAR [63]. This study suggested that memory traces in a synapse can alter signal processing in multiple adjacent connections. In addition, continuous synaptic strengthening can promote the microdomain of astrocytes to transform initially internalized (pro) brain-derived neurotrophic factor (proBDNF) into mature BDNF (mBDNF) to achieve synaptic recycling. proBDNF and mBDNF are both known to enhance TrkB signals *via* adaptive molecular mechanisms to promote the maintenance of LTP and the consolidation of memory [64].

### 3.2 Long-term Depression (LTD)

LTD refers to a long-term reduction in synaptic transmission caused by low-frequency stimuli (LFS) [65]. Astrocytes can regulate NMDAR-dependent LTD by regulating endogenous cannabinoids and D-serine. For example, Han *et al.* found that exposure to exogenous cannabis *in vivo* could activate the CB1R on astrocytes, increase the extracellular levels of glutamate, further activate the NR2B subunit of NMDAR, and promote the endocytosis of postsynaptic AMPAR. These events ultimately mediate the induction of LTD in the hippocampal CA3-CA1 synapse and participate in working memory *in vivo* [66]. The analysis of brain slices *in vitro* showed that the neuronal release of endogenous cannabinoids could activate the CB_1_R on astrocytes in the hippocampal CA1 area, induce an increase of Ca^2+^ in astrocytes, stimulate the release of glutamate, and then activate the presynaptic metabolic mGluRs to induce LTP in the CA3-CA1 synapse [59, 67].

D-serine is also known to be involved in NMDAR-dependent LTD in the hippocampus. Two recent studies support the view that the release of D-serine by astrocytes is necessary for LTD. For example, Pinto-Duarte *et al.* applied LFS (1 Hz) to induce NMDAR-dependent LTD in the Schaffer collateral. Compared with wild-type mice, astrocyte IP3R2 KO mice had impaired LTD maintenance and exhibited impairments in remote recognition memory and fear memory. The exogenous supplementation of D-serine was shown to rescue the impairment of hippocampal LTD [68]. In a second study, Best1 KO mice exhibited reduced NMDAR function, simultaneously impaired LTD, and α1 adrenergic receptor-dependent heterosynaptic LTD. These impairments could be rescued by the specific expression of Best1 in the hippocampal astrocytes or the enhancement of NMDAR function by supplementation with D-serine [52].

Navarrete *et al.* found that LFS-induced LTD in the hippocampal Schaefer collateral could enhance communications between neurons and astrocytes. Astrocytes provide sufficient glutamate at the postsynaptic membrane responsible for CA3-CA1 synaptic transmission. This mechanism requires Ca^2+^ activity in astrocytes and SNARE-dependent vesicular release. In addition, p38α mitogen-activated protein kinase (MAPK) activity in astrocytes, but not in neurons, is necessary for hippocampal LTD; the selective deletion of p38α in astrocytes blocks the expression of LTD [69]. This study challenged the view that hippocampal NMDAR-dependent LTD is reliant on direct communications between presynaptic glutamate release and postsynaptic NMDAR activation; rather, the authors proposed that the activation of astrocytes is required for LTD in the hippocampus.

Insulin-like growth factor-1 (IGF-1) is a polypeptide that has a molecular structure similar to that of insulin; this polypeptide is known to play roles in growth, development, learning, and memory [70]. Noriega-Prieto *et al.* recently found that IGF-1 induced LTP in pyramidal neurons of layer II/III of the barreled cortex in mice and that this was caused by the long-term inhibition of inhibitory synaptic transmission. Further research found that this cortical LTD was caused by the activation of astrocytes *via* the IGF-1 receptors (IGF-1Rs), thereby regulating the performance of behavioral tasks related to cortical sensory information processing [71]. Collectively, these data indicate that astrocytes also perform critical functions in LTD, either by mediating neuron-neuron communication or by directly activating postsynaptic glutamate receptors.

### 3.3 Spike-time-dependent Plasticity (STDP)

STDP is a form of Hebb long-term synaptic plasticity with rich computational properties [72]. In STDP, the order and relative millisecond time of presynaptic and postsynaptic-synaptic excitations determine the direction and amplitude of synaptic changes. Once the presynaptic neuron is stimulated for a short period of time before the postsynaptic neuron is excited, and this paired excitation is repeated multiple times, the synaptic efficacy of the synapse is enhanced in a process known as time-dependent LTP (t-LTP); time-dependent LTD (t-LTD) is induced when these events occur in the opposite order [72]. This type of plasticity requires the participation of astrocytes. For example, the activity of astrocytes is directly involved in the initiation and termination of STDP. Presynaptic t-LTD in the hippocampal CA3-CA1 synapse of mice has been shown to be converted into t-LTP as the animal develops. This form of t-LTP is expressed in the presynaptic CA3-CA1 synapse and needs to activate presynaptic mGluR5 instead of NMDARs. For this to occur, it is essential that adenosine and glutamate are released by astrocytes to activate presynaptic adenosine type 1 receptors (A1Rs) and mGluRs to mediate the conversion from presynaptic t-LTD to t-LTP [73]. In addition, adenosine and astrocytes determine the development of t-LTD in the sensory cortex. L4-L2/3 synapses in the mouse sensory cortex (S1) exhibit a presynaptic form of t-LTD from birth to the fourth week after birth; this form of t-LTD can be completely rescued by antagonizing the type 1 adenosine receptor (A1R). The adenosine-mediated loss of t-LTD is most likely induced by astrocytes [74]. In a previous study, Min *et al.* reported that in the developing cortex, endogenous cannabinoid-mediated t-LTD required Ca^2+^ signals in the astrocytes. During the induction of t-LTD, astrocytes responded to the transient increase and broadening of Ca^2+^ signals; this increase was dependent on the activation of CB1Rs [75].

### 3.4 Synaptic Remodeling

In addition to synaptic plasticity, astrocytes can also influence the formation of new synapses and the removal of old synapses. Long-term changes in synaptic efficacy are also accompanied by changes in synaptic structure, including alterations in the size of existing synapses, the formation of new synapses, and the removal of old synapses. Astrocytes are the main driving force of synaptogenesis. Thromboreactive protein (TSP) is secreted by astrocytes and is known to participate in synaptogenesis. During development, astrocytes up-regulate the release of TSP [76], which binds and activates its homologous neuronal receptor α2δ-1 to exert synaptic effects. Importantly, the TSP-mediated activation of α2δ-1 is necessary and sufficient for synaptogenesis and is also related to an increase in postsynaptic dendritic spine regeneration [77]. Although the mechanisms by which astrocytes can control synaptic formation by secreting proteins are relatively well known, the association between astrocytes and synapses remains unclear. In a recent study, Takano *et al.* used a chemical genetics method based on the cell surface fragment complementation strategy Split TurboID to identify the proteome enriched at the astrocyte-neuron junction *in vivo* [78]. These authors found that neuronal cell adhesion factor (NRCAM) is expressed by cortical astrocytes located on excitatory and inhibitory synapses and that this form of NRCAM can form complexes with gephyrin in neurons; this is a key factor in the maintenance of inhibitory synapses. In adult mice, the specific knockout of NRCAM in astrocytes was found to reduce the number of cortical inhibitory synapses; this led to a significant reduction in the inhibitory synaptic function but had little impact on excitatory synapses [78].

The phagocytosis of astrocytes is important for maintaining normal synaptic connections and plasticity in the hippocampus [79]. Astrocytes can constantly eliminate excessive and unnecessary excitatory synapse connections; this mechanism was previously verified in a *MEGF10* knockout mouse model. These mice possessed astrocytes that were deficient in phagocytosis of the *MEGF10* receptor; experiments showed that the elimination of the excitatory synapse was reduced in these mice, thus resulting in excessive and functionally impaired synaptic accumulation. Finally, *MEGF10* knockout mice showed deficits in long-term synaptic plasticity and exhibited impaired hippocampal memory formation [80].

Astrocytes can also work with microglia to promote synaptic elimination. The interleukin-1 family cytokine, interleukin-33 (IL-33), is produced by astrocytes during development. IL-33 predominantly sends signals to microglia under physiological conditions to promote the development of microglial synaptic phagocytosis and neural circuits [81]. In addition, IL-33 is known to be secreted by astrocytes to mediate synaptic steady-state plasticity in the CA1 subregion; blocking the activity of pyramidal neurons in the CA1 subregion will selectively increase the expression and secretion of IL-33 by astrocytes. Blocking IL-33 and its receptor signal transduction can inhibit the steady synaptic plasticity of hippocampal CA1 pyramidal neurons [82]. In a previous study, Koeppen *et al.* found that astrocytic ephrin-B1 acts as a regulator of adult hippocampal synaptogenesis and learning behavior. The specific deletion of ephrin-B1 in astrocytes was found to increase the density of excitatory synapses and immature dendritic spines in the mouse hippocampal CA1, thus enhancing the memory of contextual fear. In contrast, the overexpression of ephrin-B1 led to the loss of the dendritic spine and impairment of contextual memory. This study revealed that astrocytic ephrin-B1 regulates long-term memory by limiting the formation of new synapses in the hippocampus [83].

## 4 ASTROCYTES REGULATE THE ACTIVITY OF NEURAL NETWORKS

### 4.1 Network Rhythm

There are several rhythmic oscillations in the brain caused by neuronal synchronization, including delta (< 4 Hz), theta (4-12 Hz), alpha (8-12 Hz), beta (12-20 Hz), and gamma oscillations (20-100 Hz). These oscillations are closely related to the encoding, consolidation, and retrieval of memory. It has been reported that astrocytes regulate neuronal oscillations in different brain regions in a variety of ways. In the hippocampus, the activation of astrocytes can specifically reduce the power of gamma oscillations in an ATP-dependent manner [84]. In the cortical area, astrocytes contribute to the generation of slow wave oscillations (< 1 Hz) [85]. The dominant state of slow wave oscillations involves a neocortex rhythm that is characterized by synchronous neuronal discharge, a process is related to sleep and memory processes [86]. Poskanzer *et al.* found that the activation of astrocytes can regulate the transition of cortical state *in vivo*. These authors found that the Ca^2+^ activity of astrocytes was transferred to the dominant state of slow-wave oscillations prior to the spontaneous circuit and that this state of slow-wave oscillations could be induced in the local neural network *via* the optogenetic activation of astrocytes; furthermore, the activation of astrocytes could induce a local increase in extracellular glutamate, thereby increasing the firing of synergistic neurons [85]. This astrocytic input acts as a guiding synchronization signal for neurons and triggers the cortical circuit to switch to the dominant state of slow-wave oscillation.

Gamma oscillations caused by sensory inputs within different ranges of the cortical oscillation spectrum are considered to represent the foundation of cortical information processing. In a recent study, Lines *et al.* showed that astrocytes regulate sensory-induced neural network activities [24]. The response of astrocytes in the primary somatosensory cortex to sensory stimuli was dependent on an increase in Ca^2+^ signaling. Sensory stimuli have been shown to cause a transient increase in neuronal network activity within the gamma range; this was followed by a decline to the steady-state phase that occurred simultaneously with a delayed Ca^2+^ response in the astrocytes [24]. Further research showed that the activation of cortical astrocytes by specific chemical and genetic factors could reduce the gamma activity induced by sensation. However, increased levels of gamma activity were detected in transgenic mice exhibiting astrocytes with impaired Ca^2+^ activity [24]. This study showed that the activity of astrocytes was able to control the size and dynamic range of sensory-evoked gamma activity under sensory information processing.

**Fig. (2):**
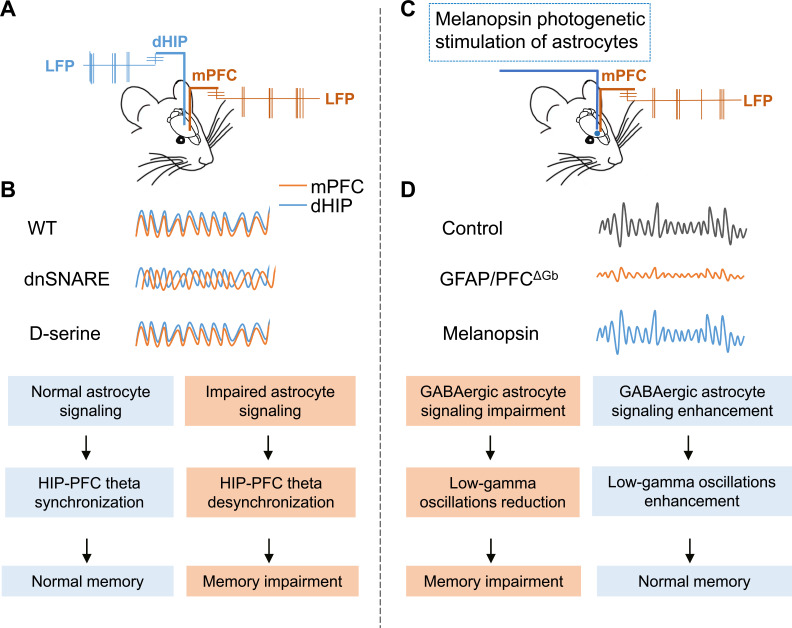
Astrocytes regulate the activity of neural networks. (**A**) Schematic diagram showing the experimental manipulation of simultaneous recordings of local field potentials (LFP) in the dorsal hippocampus (dHIP, blue) and medial prefrontal cortex (mPFC, orange); (**B**) Overlap of representative theta-filtered LFP traces in the mPFC and dHIP recorded in WT and dnSNARE and dnSNARE mice, compared to WT mice. dnSNARE mice showed reduced theta synchronization, and the intraperitoneal injection of D-serine restored theta synchronization in the dHIP-mPFC. (**C**) Schematic diagram showing the experimental manipulation of local field potential (LFP) recordings in the medial prefrontal cortex (mPFC, orange) and melanopsin optogenetics stimulation of astrocytes (blue); (**D**) Representative low-gamma (30-60 Hz specific frequency band) filtering LFP traces from control and GFAP/PFC^ΔGb^ and melanopsin transfected mice during T-maze behavioral processes.

In another study, Lee *et al.* found that astrocytes contribute to gamma oscillations and recognition memory. By performing electrocorticography recording in mice expressing tetanus neurotoxin in their astrocytes, the authors found that gamma oscillations were significantly reduced and that this was accompanied by the impaired recognition of new objects [87]. Consistent with this study, the injection of glial toxin (L-AAA) into the medial prefrontal cortex (mPFC) of experimental rats lead to a reduction in the number of astrocytes, further resulting in a reduction in gamma and other powers, and impaired cognitive flexibility [88]. In addition, signals from astrocytes are known to support theta wave synchronization in the hippocampus and prefrontal cortex; this mechanism is important for cognitive function [89]; this study used a dnSNARE mouse model to conduct electrophysiology and behavioral analysis to investigate the impact of astrocyte-derived glial transmitters on cognition (Fig. **2**). Blocking the release of glial transmitters in astrocytes was shown to trigger the basic desynchronization of theta oscillations between the dorsal hippocampus and the medial prefrontal cortex in mice. This form of desynchronization in these specific brain regions is accompanied by poor performance in cognitive tasks [89]. In contrast, supplementing D-serine was shown to restore hippocampal prefrontal theta synchronization and save spatial memory and long-term recognition memory in dnSNARE mice [89]. Finally, a recent study showed that astrocytes in the mPFC could regulate the balance between inhibition and excitability in the neural network that controls decision-making [90]. By recording the local field potential in the mPFC, researchers found that knocking out the GABA_B_ receptor (GFAP/PFC) in astrocytes in the mPFC led to a reduction in the gamma oscillation activity of cortical neurons; this also caused damage to working memory. The activation of astrocytes by Gq-coupled melanopsin was shown to restore gamma oscillation activity and working memory deficit [90]. These authors revealed that astrocytes may play a central role in controlling the inhibition of cerebral cortex circuits; furthermore, this provides a new mechanism for the processing of cortical information.

### 4.2 Neural Circuits

The human brain consists of hundreds of billions of neurons; these are interconnected to form special neural circuits, thus making the brain a powerful form of ‘biological computer’ [91]. Astrocytes are known to play an important regulatory role in the brain circuit, including different aspects of neurophysiology that are related to brain function. For example, Martin *et al.* found that astrocytes in the medial amygdala (CeM) can determine the synaptic and behavioral output of the amygdala circuit; furthermore, the chemical activation of astrocytes in the CeM can specifically inhibit excitatory input and enhance inhibitory input [92]. In addition, the latest research by Serra *et al.* identified a specific neuron-astrocyte circuit in the nucleus accumbens (NAc) [93]. These authors found that selective light stimulation of major glutamate inputs (such as the prefrontal cortex, basolateral amygdala and ventral hippocampus) induced the specific activation of astrocytic subsets in the NAc; this was inconsistent with the normal pattern of glutamate innervation, thus revealing the existence of synaptic-specific neuron-astrocyte circuits in the NAc [93]. Excitatory projections from the entorhinal cortex to the dentate gyrus (DG) are known to play an important role in the coding of memory [94]. A previous study demonstrated that the projection of the perforating pathway on hippocampal dentate granule cells (GCs) is regulated by astrocytes and that this effect is mediated by sensitive NMDAR and involves the increased release of synaptic transmitters [95]. Atypical presynaptic NMDARs (preNMDARs) can be activated by astrocytes and participate in the specific control of cortical hippocampal excitatory connections between the entorhinal cortex and the DG [96]. These preNMDARs contain a GluN3a subunit, have low sensitivity to Mg^2+^ and functionally enhance the probability of releasing medial pathway (MPP) inputs to GC dendrites. PreNMDARs also control the dynamic range of LTP in MPP-GC synapses *via* a process that requires Ca^2+^ signaling in astrocytes [96]. This study showed that this circuit-specific regulatory mechanism of astrocytes may be of great significance for changes in memory processing and pathological conditions.

**Fig. (3):**
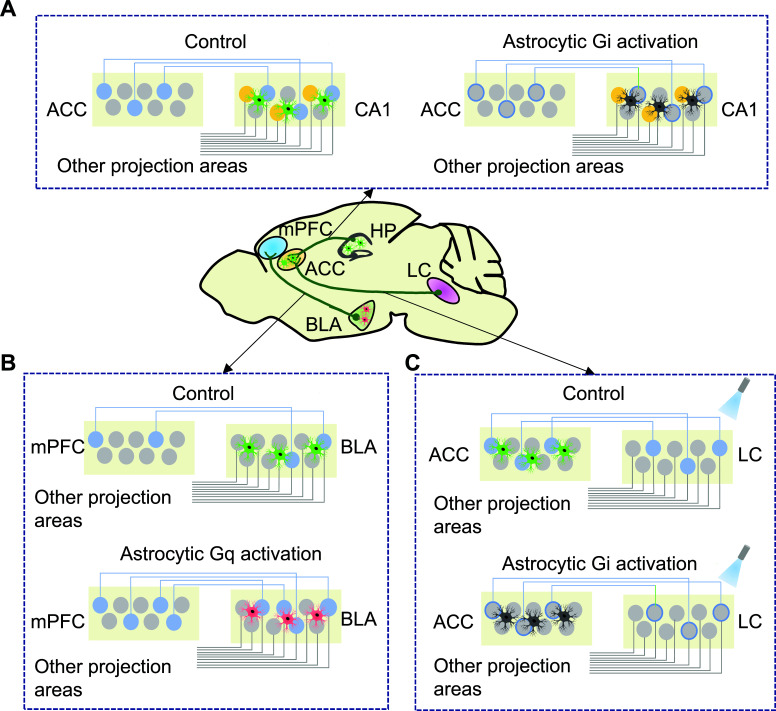
Astrocytes regulate neural circuits. (**A**) A subpopulation of pyramidal neurons (blue) in the hippocampal CA1 area projects to the ACC, and hippocampal CA1 area astrocytes (green) regulate the activity of this subpopulation, thus allowing ACC neuronal labeling, a process critical for remote and schema-associated memory. In contrast, the Gi activation of astrocytes (gray) in the hippocampal CA1 area specifically inhibits ACC projections to CA1 neurons during memory formation, resulting in the impaired formation of remote memory. Recent memories may be mediated by CA1 neurons that do not project to the ACC (orange). (**B**) Gq activation of BLA astrocytes (red) specifically enhances communication between the BLA-MPFC and increases auditory cue fear memories. (**C**) A subpopulation of LC neurons projecting to the ACC is critical for aversive learning and memory, and the optogenetics activation of LC neurons projecting to the ACC enhances aversive memory formation. However, when astrocytes are activated by Gi (gray), there is a disruption in the optogenetics process of LC neuron recruitment to the ACC, thus inhibiting the enhancement of aversive memory.

Compared with neurons, astrocytes do not have long-distance connections and are considered to function locally. However, recent studies highlight the fact that astrocytes may regulate long-distance neurons *via* gap junction-related astrocyte networks. For example, Zhao *et al.* found that the optogenetic activation of astrocytes in the left primary motor cortex of mice could alleviate kainic acid-induced seizures on the right side of the brain and also influenced the electrical activity of neurons in the right primary motor cortex in which more than 80% of high-frequency (> 5 Hz) firing pyramidal neurons were suppressed [97]. In addition, astrocytes can exert an important impact on the long-distance projection of neurons. For example, Kol *et al.* reported that astrocytes promote the formation of remote memory by regulating hippocampal cortical communication during learning [98]. By specifically expressing the Gi-coupled design receptor hM4Di in hippocampal CA1 astrocytes, the activation of astrocytes during learning was shown to damage remote memory rather than short-term memory and reduced activity in the anterior cingulate cortex (ACC). In addition, Gi-induced activation of astrocytes was reported to destroy the communication links between CA3 and CA1, weaken the activation of ACC neurons induced by training, and damage remote memory [98]. Astrocytes not only regulate neural activity locally (at the synaptic level); they also regulate neural activity remotely *via* neural circuits. Schema memory is a concept of cognitive psychology and represents an interactive knowledge framework that is stored in long-term memory in the form of a network structure [99]. A recent study reported that astrocytes regulated the establishment of schema memory in rats *via* the hippocampal CA1-ACC circuit [100]. These authors used a behavioral paradigm of food location pairing (PAs) to dynamically record Ca^2+^ signals in the CA1-ACC projection neurons and ACC neurons during the formation of schema memory. This strategy revealed the influence of chemical activation in the CA1 astrocyte-Gi pathway on the CA1-ACC network at three stages during the formation of schema memory. Analysis showed that the activation of astrocytes in the CA1 before training led to a failure to form an effective schema memory. In addition, new information relating to Pas could not be absorbed quickly. The imaging of Ca^2+^ signaling by fiber optic recordings showed that activation of the Gi pathway in CA1 astrocytes reduced the expression of c-FOS transmitted from CA1 to ACC neurons at the beginning and consolidation stages of task learning. Furthermore, during the consolidation stage, this process also reduced the activity of ACC neurons downstream, thus interfering with the establishment of schema memory. When schema memory has been fully established, activation of the Gi pathway in astrocytes no longer affects the activity of ACC neurons [100]. In another study, Lei *et al.* showed that the chemical activation of astrocytes in the basolateral amygdala participated in the formation of fear memory by regulating communication between the amygdala and the prefrontal cortex [101]. Furthermore, these authors found that the Gq-induced activation of astrocytes during fear learning increased the expression of c-FOS in the basolateral amygdala (BLA) and the mPFC during the fear-conditioned reflex. In addition, retroviral tracking results showed the activation of astrocytes by Gq-induced projection specificity in BLA-mPFC neurons during fear learning. Moreover, electrophysiology recording demonstrated that the activation of Gq signals in astrocytes in the BLA could promote the connection of field potential and phase locking value between the BLA and the mPFC (Fig. **3**) [101].

## 5 THE FUNCTIONAL ROLE OF ASTROCYTES IN MEMORY

### 5.1 Spatial Memory

In a previous study, Goshen *et al.* used a two-photon microscope to image the activity of astrocytes in the hippocampal CA1 and found that astrocytes could encode position-related information in the spatial environment [102]. In a familiar environment, astrocytes exhibited continuous enhancement activity in response to reward, but not in the new environment. After learning a new environment or receiving a new reward in a specific environment, the Ca^2+^ activity of astrocytes increases. These researchers also built a linear regression decoder for each mouse and found that it was possible to predict the position of mice in a familiar environment based on the activity of astrocytes [102]. In addition, Curreli *et al.* conducted two-photon calcium imaging in astrocytes in the hippocampi of mice that navigated in a virtual space and found that the Ca^2+^ signaling of astrocytes in the hippocampus encoded spatial information. In addition, the spatial information encoded by astrocytes was complementary and synergistic with the spatial information carried by neurons. The combination of astrocytic and neuronal signals produced further information relating to an animal’s position [103]. This hippocampal functional model assumed that information relating to external environmental variables is crucial for spatial navigation and memory and was encoded only within the neuronal population [104, 105]. These results challenged the established viewpoint, indicating that there may be novel and unexpected cellular mechanisms involved in how brain circuits encode information.

The activation of Gi or Gq GPCR signals *via* hM4Di and hM3Dq DREADDs or Gq-coupled melanopsin is commonly used to stimulate Ca^2+^ dynamics in astrocytes. For example, after specifically inhibiting Gq GPCR signals in astrocytes, researchers found that the spatial working memory of experimental mice was damaged [106]. Gene knockout techniques and pharmacological studies have also proved that astrocytes participate in the regulation of spatial memory. For example, connexin 30 (Cx30) and connexin 43 (Cx43) mediate the coupling of astrocytes to form a large intercellular network [107]. The double knockout of Cx30 and Cx43 in astrocytes destroyed the coupling between astrocytes and led to the extensive activation of both astrocytes and microglia. Furthermore, the excitability, excitatory synapse transmission, and LTP of hippocampal CA1 neurons were also affected in these transgenic mice. In addition, the mice exhibited sensory motor defects and a complete lack of spatial learning and memory [108]. In addition, inhibiting the Cx43 half channel (Cx43hc) in the prefrontal cortex was also shown to damage the spatial working memory of experimental rats [109]. Lipopolys-stimulated lipoprotein receptor (LSR) is one of the lipoprotein receptors in the CNS and plays an important role in maintaining cholesterol homeostasis in the brain [110]. In a previous study, El Hajj *et al.* established a Glast Cre lsrfl/fl conditional KO mouse model to trigger the specific loss of LSR in astrocytes and then assessed the spatial working memory of these mice. These authors found that the spatial working memory was damaged in mice possessing astrocytes in which the LSR had been conditionally knocked out [111]. Mitochondrial superoxide dismutase (SOD2) is an antioxidant that is present in the mitochondrial matrix and can detoxify superoxide production during mitochondrial respiration and maintain mitochondrial homeostasis. The specific reduction of SOD2 in astrocytes caused damage to the hippocampus-dependent spatial working memory of male mice but did not affect the learning and memory of female mice [112]. The *GDI1* gene encodes αGDI, a protein that controls the small GTPase cycle and is thought to be involved in vesicular transport. Mutations in the human *GDI1* gene have been shown to lead to intellectual impairment [113, 114]. The knockout of *GDI1* in mouse astrocytes was previously shown to impair spatial working memory [115]. As a factor secreted by astrocytes, IL-33 is known to play an important role in the synaptic steady-state plasticity of the adult hippocampus. IL-33 promotes the formation of hippocampal excitatory synapses and neuronal transmission in both *in vitro* hippocampal cultures and in adult mice *in vivo*; blocking the signal transduction of IL-33 inhibited the steady-state synaptic plasticity of CA1 pyramidal neurons and caused damage to the formation of spatial memory (Table **1**) [82].

### 5.2 Aversive Memory

Astrocytes are also involved in the formation of fear memory. For example, the chemical, genetic or optogenetic Gq activation of hippocampal CA1 astrocytes during memory acquisition in mice was shown to promote the formation of contextual fear memory [116]. In the process of fear memory consolidation, the optogenetics activation of hippocampal astrocytes can reduce fear memory and improve anxiety-like behaviors [117]. Similarly, Gq activation in astrocytes of the medial central amygdala was shown to lead to the disappearance of fear memory in the fear-conditioned reflex paradigm [92]. The chemogenetic activation of astrocytes in the BLA can promote the formation of cued fear memory [101]. These contradictory results indicate that astrocytes in different regions of the brain, or in the same region, might play different roles in different processes of learning, and memory. The normal activity of Rac1 in astrocytes is necessary for the activation of neurons and the formation of memory. The activation and inactivation of Rac1 activity in astrocytes of the BLA has been shown to reduce the excitability of neurons, thus impairing the acquisition of fear memory [118]. In addition, the expression of the cholinergic muscarine 1 receptor (Chrm1) in astrocytes of the hippocampal DG region can regulate the formation of contextual fear memory. The knockout of Chrm1 in astrocytes leads to the impairment of contextual fear memory in mice; furthermore, the overexpression of Chrm1 was shown to rescue the impairment of contextual fear memory in mice [119]. A recent study used electron microscopy to investigate changes in the synaptic coverage of hippocampal astrocytes during encoding and the consolidation of fear memory in mice. These authors found that the encoding and consolidation of contextual fear memory was accompanied by the transient contraction of astrocyte lobules from the synaptic gap and the increased activation of NMDA receptors [120]. In the auditory cortex, fear stimulation activates α7-nicotinic acetylcholine receptors (nAChRs) in the subpopulation of astrocytes in the auditory cortex; knocking out these α7-nAChRs significantly impels the persistence of fear memory [121]. The authors of this study suggested that astrocytes are not only involved in the dynamic regulation of neuronal responses but also play a key role in the long-term storage of information in the brain.

Stress can cause the release of glucocorticoids (GCs), which regulate energy metabolism and play a role in emotional memory. Astrocytes express glucocorticoid receptors (GRs); the selective knockout of GRs in astrocytes impaired the expression of aversive memory in two paradigms of Pavlov’s conditioned reflex: the contextual fear-conditioned reflex and conditioned aversion [122]. Pain is a conscious subjective experience, usually caused by pain stimuli, involving both sensory and emotional factors. Iqbal *et al.* previously reported that astrocytes in the ACC regulated the visceral pain aversion memory of rats *via* L-lactic acid signaling [123]. These authors reported that when pain aversion memory was formed in rats, a large amount of L-lactic acid was released from ACC. In contrast, blocking glycogen decomposition in the astrocytes of the ACC reduced the levels of L-lactic acid and destroyed the formation of aversion memory. The injection of exogenous L-lactic acid reversibly transformed the pain aversion memory disorder in experimental rats. In addition, the optogenetic activation of astrocytes in the ACC promoted the release of L-lactic acid and enhanced the formation of pain-related aversion memory. However, short-term activation of the Gi pathway in astrocytes of the ACC prior to conditioned place avoidance training reduced the level of lactate and inhibited pain-related aversion memory [123]. Norepinephrine signaling in astrocytes of the ACC has been reported to play a role in the formation of pain-related aversion memory in rats [124]. The authors of this study reported that actinogenesis activated β2ARs receptors on the astrocytes in the ACC to promote aversive memory and induce learning-dependent plasticity. The specific knockout of β2ARs was shown to inhibit aversive learning and memory. The Gi-induced activation of astrocytes was shown to destroy the aversive memory induced by optogenetic activation of neurons in the locus coeruleus (LC) projecting into the ACC [124].

### 5.3 Recognition Memory

Recognition memory, the ability to retain and recognize stimuli or events, is the foundation of daily life and survival. In a previous study, Cheung *et al.* reported the development of a fluorescent probe that can track glutamine in living cells. By using this probe on slices of mouse hippocampi, these authors found that the Cx43hc of astrocytes mediated the activity-dependent transfer of glutamine from astrocytes to synapses. By performing new object recognition experiments, the authors found that inhibiting the function of the Cx43hc led to the impairment of recognition memory in experimental mice. However, mice administered with glutamine did not exhibit memory impairment. This study showed that the formation of new recognition memory in adult mice requires glutamine mobilization in astrocytes [125]. The barrel cortex, as a major component of the surface sensory cortex, has a one-to-one correspondence between each functional column in its fourth layer and the facial antennae of rodents. The neurons in each functional column mainly receive incoming information from the corresponding main antennae and produce evoked responses. In a previous study, Noriega-Preto *et al.* found that the loss of specific IGF-IR in barrel cortical astrocytes resulted in impairment in the recognition task [71]. In another study, Robin *et al.* reported that the loss of the CB1 receptor in astrocytes led to the impairment of recognition memory in GFAP-CB1-KO mice. Furthermore, the impairment of recognition memory in GFAP-CB1-KO mice was improved by the exogenous supplementation of D-serine or blocking D-serine catabolism with drugs [60]. Bmal1 is a transcription activator and is considered as the main driving factor of the mammalian biological clock [126]. A previous study found that deletion of the core clock gene *Bmal1* in astrocytes led to the destruction of short-term recognition and spatial memory, while pharmacological regulation of the GABA receptor signaling pathway could completely reverse this behavioral defect [127].

## 6 THE ROLE OF ASTROCYTES IN MEMORY-RELATED DISEASES

AD is characterized by memory impairment and the presence of reactive astrocytes surrounding the amyloid plaques [128]. However, a key question is whether these reactive astrocytes can cause memory impairment. Aging is a recognized risk factor for most neurodegenerative diseases. In addition to neurons, astrocytes also play an important role in the process of brain aging [129]. The main symptoms of PTSD include pathological fear memory enhancement, which manifests as the recurrence or flashback of traumatic memory and avoidance. Neurons, as well as glial cells, are known to be involved in the regulation of the stress response [130]. Chronic traumatic stress can also cause structural atrophy of astrocytes [131]. Although neuronal abnormalities are considered to be the main cause of memory disorders, astrocytes undoubtedly also play a critical role in this pathological process, either by regulating neurons or by self-regulation. Next, we discuss the critical roles of astrocytes in memory-related disorders.

### 6.1 AD

Previous research showed that reactive astrocytes produced the inhibitory transmitter GABA in a manner that was dependent on monoamine oxidase B (MAO-B). The authors found that the GABA released from astrocytes activated neuronal GABA_A_ and GABA_B_ receptors, thereby inhibiting synaptic transmission and impairing synaptic plasticity and memory [132]. In addition, the excessive production of hydrogen peroxide by severely reactive astrocytes was shown to trigger neurodegeneration in APP/PS1 mice [133]. This finding provided profound insight into the current theory for the pathogenesis of AD. In patients with severe AD, immune therapy against the β-amyloid protein (Aβ) did not delay neurodegeneration and cognitive decline, further emphasizing the pathological role of reactive astrocytes [133].

Astrocytes are known to release GFAP upon activation. Plasma GFAP is an early marker of amyloid protein in AD. By assessing the relationship between GFAP and amyloid protein in the body, researchers discovered that the plasma concentrations of GFAP in the positive group of patients were significantly higher than patients in the amyloid pathology group [134]. In addition, when investigating the correlation between blood GFAP and memory in AD patients, researchers found that higher GFAP levels were associated with lower memory scores; consequently, an increase in GFAP, a marker of astrocyte activation, may reflect a decline in memory function [135].

The accumulation of Aβ and phosphorylated tau are common pathological features of AD. High levels of aquaporin-4 (AQP4) in astrocytes are known to facilitate the clearance of soluble Aβ from the brain parenchyma along the paravascular pathway [136]. In contrast, the knockout of AQP4 enhanced the production of Aβ in the brains of APP/PS1 mice [137]. In another study, Du *et al.* found that the downregulation of hippocampal GluN2A in astrocytes could aggravate Aβ-induced spatial memory impairment [138]. Astrocytes possess a unique non-circulating metabolic pathway for urea. Upon commencing Aβ treatment, astrocytes begin to cycle urea, thus eliciting an increase in the levels of aspartic acid and putrescine; these can induce the impairment of memory [139]. Importantly, tau was found to accumulate in astrocytes of the hippocampal DG region in AD patients [140]. In a mouse model, the overexpression of tau in astrocytes can lead to alterations in mitochondrial dynamics and functionality, thereby inducing neuronal dysfunction and memory deficits [140].

Lipid imbalance is an important feature of several neurodegenerative diseases, especially in AD. *APOE4* is a known risk gene for AD [141]; a previous study reported that astrocytes are the major cell type that produces the most APOE [142]. APOE4 can cause degenerative changes in the pericytes of capillaries in the brain, thereby accelerating the destruction of the blood-brain barrier (BBB). Impairment of the BBB makes it easier for toxic substances in the blood to enter the brain, thus leading to the cognitive decline of APOE4 carriers [143]. In addition, APOE4, rather than APOE3, can activate the CypA-MMP9 pathway in cerebrospinal fluid, thus accelerating the breakdown of the BBB and leading to neuronal and synaptic dysfunction [143]. In addition, APOE4 was shown to destroy the lipid homeostasis of astrocytes derived from human induced pluripotent stem cells (iPSC). Human astrocytes with the APOE4 genotype are known to contain a large number of neutral lipids and cholesterol; these astrocytes can accumulate a large number of lipid droplets, in which unsaturated fat acid chains can lead to an imbalance in the lipid status of astrocytes [144]. Gliican-4 (GPC-4) is a protein secreted by astrocytes and regarded as the binding partner of APOE4 to drive the hyperphosphorylation of tau [145]. In addition, researchers recently discovered that human-specific APOE4 drove disorders of lipid metabolism in astrocytes and microglia, thus increasing the risk of AD [146]. In addition, the selective removal of APOE4 from astrocytes was shown to reduce Tau-mediated neurodegeneration [147].

**Fig. (4):**
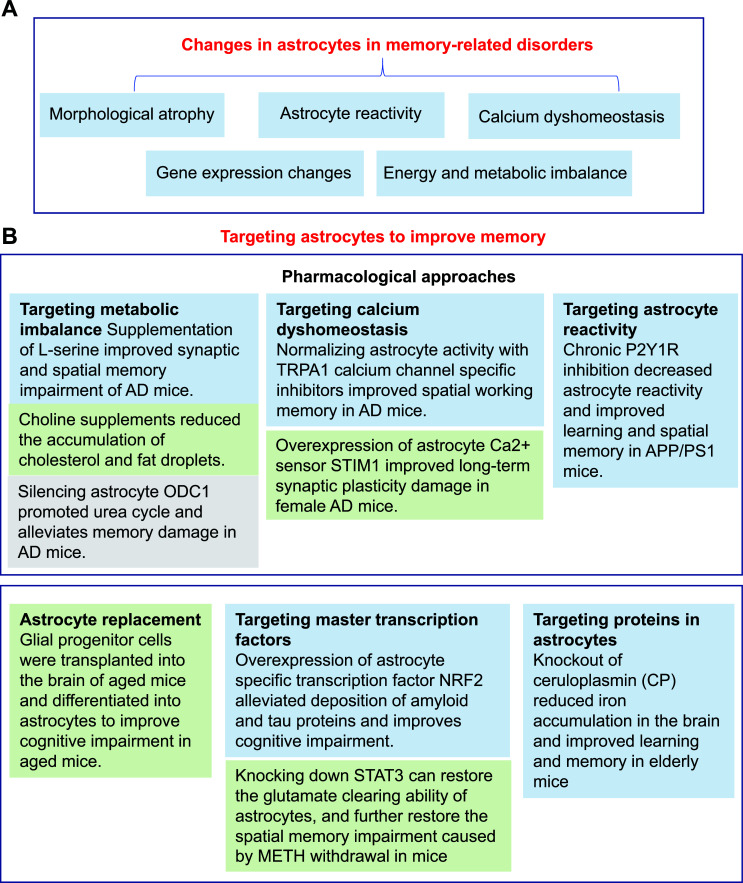
The role of astrocytes in memory-related disorders. (**A**) Astrocytes exhibit alterations in memory-related disorders in several aspects: morphological structure, the acquisition of a reactive phenotype by astrocytes, dysregulation of calcium homeostasis, alterations in gene expression, and metabolic imbalances. (**B**) Based on experimental evidence, several strategies, including pharmacological approaches, have been proposed to target astrocytes to improve memory.

Based on basic experimental evidence, targeting astrocytes might improve the memory impairment of AD (Fig. **4**). For example, the oral application of L-serine was shown to prevent the synaptic and spatial memory impairment of a mouse model of AD [148]. L-serine, the precursor of D-serine, is produced by glycolysis in the astrocytes. The impaired synthesis of L-serine is known to aggravate the cognitive impairment of AD. Moreover, AD mice were shown to exhibit a low level of occupancy in co-agonist sites for NMDAR. The oral application of L-serine was shown to improve the impairment of synaptic function and spatial memory in AD mice [148]. APOE4 is one of the strongest genetic risk factors for late-onset AD [145]. In a previous study, Sienski *et al.* found that the additional supplementation of choline, a dietary supplement that is safe for human beings, could reverse the damage caused by APOE4 in astrocytes [144]. A recent study found that ornithine decarboxylase 1 (ODC1) plays a key role in separating the detoxicated urea cycle and the degradation pathway for putrescine, a toxic by-product. Silencing ODC1 in astrocytes promotes the transformation of ornithine to putrescine and reduces the production of putrescine and GABA in a mouse model of AD, thus reducing memory impairment in AD patients [139]. Previous research found that the Aβ-dependent transient receptor potential A1 (TRPA1) calcium channel could trigger the excessive activity of hippocampal astrocytes and then induce the excessive activity of nearby neurons [149]. In this regard, a recent study found that the blockade of the TRPA1 calcium channel could normalize the activity of astrocytes, help to maintain the integrity of synaptic structure and improve spatial working memory in a mouse model of AD [150]. In addition, the overexpression of the astrocytic Ca^2+^ sensor STIM1 was shown to rescue LTP impairment in female mice with AD [151]; in addition, astrocytes were shown to exhibit impaired Ca^2+^ activity in PS2/APP mouse models of AD. This low level of Ca^2+^ activity was associated with the reduced storage of Ca^2+^ and the downregulation of STIM1. Moreover, reduced Ca^2+^ activity in astrocytes has been shown to lead to long-term impaired synaptic plasticity in PS2APP mice. In addition to improving impaired tactile recognition memory, the overexpression of STIM1 in astrocytes also rescued Ca^2+^ activity in astrocytes and synaptic plasticity [151]. The overactivity of astrocytes is an important factor in the abnormal functionality of neural networks in AD. Research showed that long-term treatment with a P2Y1 purine receptor (P2Y1R) antagonist reduced reactivity in astrocytes and normalized astrocytic dysfunction and neural networks, thus improving network dysfunction and spatial memory impairment in a model of AD [152].

A pertinent question at this point is whether astrocytes are able to clear the accumulation of β-amyloid protein and tau protein to improve the symptoms of AD. A recent study found that the combination of gemfibrozil (GFB) and retinoic acid (RA), a drug used to treat high cholesterol, could activate PPARα and then enhance the effect of astrocytes on Aβ absorption and the degradation of amyloid protein, thus reducing brain Aβ burden, improving spatial learning, and memory in the 5 x FAD mouse model of AD [153]. Furthermore, a recent study showed that the programming of microglia with astrocyte-derived interleukin-3 (IL-3) improved pathological effects and cognitive function in AD [154]. These researchers found that IL-3 was mainly derived from a subpopulation of astrocytes in the mouse brain and that Il3^−/−^5xFAD mice suffered from short-term and spatial memory impairment following IL-3 knockout. As AD progresses, microglia increase their sensitivity to IL-3 by expressing IL-3Rα; the IL-3 induces extensive programming of the microglia transcriptome, deploys immune and motor responses, and promotes microglial immune activation, and the aggregation and clearance of Aβ and tau aggregates [154]. During the development of AD, astrocytes exhibit extensive changes at the transcription and protein levels. This generates the question as to whether the symptoms of AD could be improved by targeting specific key transcription factors. Jiwaji *et al.* showed that the overexpression of Nrf2, an astrocyte-specific transcription factor, reduced the accumulation of Aß-deposition and phosphorylated tau and rescued transcriptional disorders in the brain and the damage caused to fear memory [155].

### 6.2 Brain Aging

The morphology and functionality of astrocytes are known to change remarkably in aging mice; this is manifested by the gradual atrophy of morphology, the significant reduction of astrocyte-astrocyte coupling, the deficiency of K^+^ clearance and glutamate recovery, and the spatiotemporal reorganization of Ca^2+^ events [156, 157]. In addition, aging can change the spontaneous Ca^2+^ activity of astrocytes in the subcellular domain. Further evidence suggests that the reduction of Ca^2+^ activity in the endfeet of astrocyte is related to a reduction in AQP4 expression [158].

In fact, aging may have a profound impact on the functionality of astrocytes. For example, the expression patterns of specific genes in astrocytes were shown to change with age, while the patterns of gene expression in neurons were only mildly affected [159]. In addition, the astrocytes of aged mice are known to exhibit A1-like reactivity, while A1-type astrocytes were demonstrated to lose normal functionality when compared with normal astrocytes, such as the production of complement components and strong neurotoxicity [160, 161]. Other research showed that senile astrocytes accumulated in the brain and showed reduced functionality and the secretion of senescence-associated secretory phenotype factors, which are known to contribute to neuroinflammation and neurotoxicity [162, 163]. By analyzing the transcriptome of astrocytes in aged mice, researchers demonstrated that the genes that are upregulated in astrocytes are related to the immune signaling pathway and synaptic elimination [164, 165]. Excitatory amino acid transporter 2 (EAAT2) is the main glutamate transporter in the brain and is predominantly expressed in astrocytes [166]. Sharma *et al.* further showed that the lack of EAAT2 in astrocytes rather than neurons in the hippocampus concurred with the gene expression profiles associated with human aging and AD. In addition, the lack of EAAT2 in astrocytes is known to accelerate age-related cognitive deficits [167].

The functionality of astrocytes in old animals is known to be abnormal. This suggests that it may be possible to reverse the aging process in the brain by transplanting young and healthy astrocytes. Yang *et al.* found that the transplantation of glial progenitor cells derived astrocytes into the cerebral cortex of adult mice improved the microenvironment of the brain in aging mice, promoted neuronal survival, and restored memory [168]. In another study, Xu *et al.* found that Yes-associated Protein (YAP) prevented the premature aging of astrocytes and the cognitive decline of Alzheimer's disease by regulating the cyclin-dependent kinase 6 (CDK6) signaling pathway [169]. These authors found that YAP was down-regulated and inactivated in a Hippo pathway-dependent manner in the hippocampal astrocytes of aging mice and a mouse model of AD, as well as in aging astrocytes induced by D-galactose and paraquat. The conditional knockout of YAP in astrocytes was shown to promote the aging of astrocytes, both *in vivo* and *in vitro*. Furthermore, the heterotopic overexpression of CDK6 was shown to prevent the aging of astrocytes caused by YAP knockout, at least in part. In addition, previous research showed that YAP signaling was activated by Xmup-1, an inhibitor of Hippo kinase MST1/2 and that the aging of astrocytes was delayed *in vivo*, thereby improving the cognitive function of elderly mice and mice with AD [169].

Research has also shown that the levels of miR-335-3p were increased in aged astrocytes in culture and in aged hippocampi. The impairment of cholesterol synthesis in astrocytes caused by the overexpression of miR-335-3p down-regulated the synaptic protein PSD95 in neurons while reducing the levels of miR-335-3p in the aged mouse hippocampus increased the levels of PSD95 protein and the production of cholesterol [170]. Copper blue protein (CP) is an iron oxide enzyme that plays a crucial role in maintaining iron homeostasis [171]. Li *et al.* demonstrated that the knockout of CP in astrocytes reduced age-dependent iron accumulation in the brain and improved the learning, and memory functions of elderly mice [172].

### 6.3 PTSD

Previous research showed that chronic restraint or traumatic stress can cause damage to hippocampal glia, especially astrocytes and that these events are related to emotional and cognitive impairment [173-176]. In addition, by utilizing the stress-enhanced fear learning (SEFL) model, researchers found that the stress-induced IL-1 signal in the dorsal hippocampus, which is crucial for the development of SEFL, and astrocytes were the main source of IL-1 production [12]. Hippocampal atrophy or impaired hippocampal function is one of the most common morphological changes reported in PTSD patients [177, 178]. In an animal model of PTSD, hippocampal atrophy is caused by the loss of astrocytes, at least in part [175, 179]. A recent proteome-wide association study of PTSD revealed that 11 genes were involved in the pathogenesis of PTSD, several of which were preferentially expressed not only in neurons but also in astrocytes and other glial cells [180].

The extinction of fear memory is an important therapeutic target for PTSD [181-183]. Previous research showed that cotinine promoted the extinction of fear memory in rodents and that the infusion of cotinine into the medial prefrontal cortex increased the survival of astrocytes [179, 184]. Kir4.1 is an inward rectifying K^+^ channel expressed only in glial cells and is considered a potential therapeutic target for mental disorders [185, 186]. In lipopolysaccharide (LPS) or single prolonged stress (SPS) mouse models, the expression levels of Kir4.1 in hippocampal astrocytes were significantly increased, while the reduction of Kir4.1 in the hippocampus promoted the extinction of fear [187]. Fibroblast growth factor 2 (FGF2) is a multifunctional growth factor that is essential for the development of the CNS [188]. Research has shown that intraperitoneal injection of FGF2 can block the SPS-induced PTSD fear response and anxiety behavior *via* astrocytic but not neuronal mechanisms [189]. Further analysis revealed that FGF2 restored glutamate uptake in astrocytes *via* the JAK/STAT pathway and improved fear and anxiety-like behavior in a rat model of PTSD [190].

### 6.4 Addiction

Astrocytes are believed to be involved in the development of drug addiction [191-193]. Cannabis-induced memory impairment is also known to correlate with the functionality of astrocytes. Delta-9 tetrahydrocannabinol (THC), a component of cannabis, is considered to be a partial agonist of cannabinoid receptor 1 in the brain. Acute exposure to THC is known to cause damage to working memory [66]. Hippocampal astrocytes express endogenous schizophrenia dominant negative disruption protein 1 (DN-DISC1), and exposure to THC during puberty was shown to synergistically affect the recognition and memory of adult mice [194]. Cocaine-related memory is an important source of desire, and interferes with drug withdrawal ability. Reducing the intensity of clue drug memory by extinction is of therapeutic value for the treatment of cocaine addiction. In a previous study, Shelkar *et al.* found that the selective ablation of the GluN1 subunit in astrocytes of the nucleus accumbens promoted the extinction of cocaine reward memory [195]. In addition, destroying the production of lactic acid by astrocytes temporarily impaired the preference for cocaine [196]. Methamphetamine (METH) is a common substance of abuse. Recent research demonstrated that METH withdrawal caused damage to the spatial memory of mice and led to an increase in glutamate levels in the hippocampal CA. Following METH withdrawal, astrocytes exhibited impaired glutamate clearance ability, as demonstrated by the increased expression of transcription activating factor 3 (p-STAT3) in astrocytes. By selectively knocking down STAT3, astrocytes in the hippocampal CA1 were able to recover their ability to clear glutamate and improve the damage to spatial memory caused by METH withdrawal in experimental mice [197].

## 7 FUTURE PROSPECTS

Astrocytes are considered to be a homogenous cell population, and it is generally assumed that astrocytes from different brain regions are functionally interchangeable. However, recent studies have proven that astrocytes, like neurons, are heterogeneous. Astrocytes are distributed in various brain regions, exhibit regional specificity and can distinguish between different neuronal subtypes and regulate their activities [1, 10, 198]. Astrocytes have different effects on neurons according to their genetic characteristics. For example, Huang *et al.* used the recently developed Aldh1l1-CreER mouse and specifically knocked out the NFIA transcription factor. Then, the authors analyzed changes in four brain regions, including the olfactory bulb, cortex, hippocampus and brain stem, to identify changes in the morphological, physiological and gene expression characteristics of astrocytes. Surprisingly, astrocytes in the hippocampus showed significant changes but not the other three regions. Astrocytes in the hippocampus exhibited lower levels of calcium activity, a weaker ability to detect neurotransmitters, and reduced tightness in the connections with neurons. All of these changes in morphology and function were related to learning and memory [53]. Chrdl1 is highly enriched in cortical astrocytes and is known to regulate synaptic function [199]. Although significant progress has been made, there are still many challenges that need to be resolved. For example, we are lacking specific techniques to better understand the intrinsic characteristics of astrocytes. Moreover, the local circuits and behavioral specificity of neurons and the astrocytes that interact with neurons still need to be clarified. This requires the development of better astrocyte-specific research tools, technologies and experimental designs to resolve the remaining challenges and bridge the gaps in our existing knowledge.

A deeper understanding of the brain will stimulate new types of biological computing and artificial intelligence. For example, a recent study selected two spiking neural networks, cortical spiking networks (CSNs) and cortical neuron-astrocyte networks (CNANs), to investigate these brain-inspired networks from a learning perspective and found that CNANs provided more powerful information encoding functions than CSNs [118]. Furthermore, the transfer of information from the CNANs to the CSNs could improve the recognition ability of CSNs without relying on a time-consuming training process [200]. Moreover, computational models also provide evidence and understanding of the role of astrocytes in the processing of memory in the brain. Recently, researchers proposed a neuron-glia network model and used this model to demonstrate that interactions between synapses and astrocytes can produce various forms of working memory [201]. In addition, a synaptic attractor model of working memory has also been developed and used to investigate the role of astrocytes in the regulation of working memory [202]. These authors found that astrocytes were key determinants for the duration of working memory in this model and that astrocyte signals can promote top-down volitional control of different mechanisms of working memory representation and duration [202].

With the development of viral vector technologies such as adeno-associated viruses (AAVs) and retroviruses, rapid progress has been made in the field of cell reprogramming [203]. Moreover, research has proved that astrocytes have the potential to be reprogrammed. For example, Xiang *et al.* showed that overexpression of the *NeuroD1* gene *via* an AAV vector could transform astrocytes in the brain into neurons [204]. In addition, Zhang *et al.* demonstrated that overexpression of transcription factor *DLX2* could transform mature astrocytes in the brains of mice into induced neural progenitor cells, which subsequently differentiated further into neurons, astrocytes and oligodendrocytes [205]. In another study, a retrovirus was used to overexpress the *NeuroD1* transcription factor in the cortex of a mouse model of AD; this study clearly demonstrated that astrocytes could be reprogrammed into glutamate neurons, which were subsequently integrated into local neural circuits [206]. However, it remains unclear as to whether the reprogramming of glial cells into neurons can rescue cognitive deficits in AD mouse models.

Astrocytes not only influence synaptic plasticity in neurons; they also regulate neural circuits. Importantly, astrocytes may also represent a potential therapeutic target for memory-related neurological diseases. Our understanding of the involvement of astrocytes in memory is still incomplete, and more research is needed to confirm existing findings and better elucidate the specific role played by astrocytes in memory processes. For example, there are still many memory-related astrocyte activities that are not fully understood during the acquisition and storage of information. This is because it is difficult to identify well-defined astrocytes *in vivo* and monitor their activity in animal models during different learning stages and different learning tasks over several days. Moreover, the diversity of signal pathways by which astrocytes influence neuronal signaling, the many dimensions of memory, as well as the diversity of memory mechanisms all represent major challenges if we are to gain a comprehensive understanding of the function of astrocytes in memory. In addition, although recent evidence suggests that astrocytes may serve as a pathogenic and treatment target for memory disorders, this needs to be confirmed in future research involving specific manipulation by genetic methods.

## CONCLUSION

In conclusion, this review provides important reference guidelines relating to our current understanding of memory and memory-related disorders from the perspective of astrocytes. Targeting astrocytes might represent a potential strategy with which to treat memory-related neurological diseases.

## Figures and Tables

**Table 1:** The roles of astrocytes performed in memory.

**Memory** ** Types**	**Manipulation or Detection Methods**	**Proposed Mechanism**	**Outcomes**	**References**
Spatial memory	Long-term imaging of hippocampal CA1 astrocyte by two-photon microscopy	Calcium kinetics of astrocyte	Astrocytes participate in encoding spatial memory	[102]
	Simultaneous calcium imaging of hippocampal astrocytes and neurons by two-photon microscopy	Calcium kinetics of astrocyte	The calcium signal of astrocytes encodes spatial information, which is complementary to the spatial information encoded by neurons	[103, 106]
	β-adrenergic receptor kinase 1 specifically inhibits the Gq GPCR signal of astrocyte	Gq GPCR signaling	Impairment of spatial memory	[108]
	Astrocyte-specific Cx30 and Cx43 double knockout	Synaptic transmission and LTP	Impairment of spatial memory	[109]
	Pharmacological blockade of the CX43 half channel in astrocytes in PFC	Synaptic transmission	Impairment of spatial memory	[111]
	Specific Knockout of Lipoprotein Receptor LSR in astrocyte	Cholesterol metabolism	Impairment of spatial and working memory	[112]
	Selective ablation of astrocyte SOD2	Mitochondrial homeostasis, D-serine and LTP	Hippocampus-dependent spatial working memory in male mice	[115]
	*Gdi1* gene deletion in astrocyte	Glycometabolism	Impairment of spatial memory	[82]
	Inhibition of IL-33/ST2 signaling in astrocyte	Homeostatic synaptic plasticity	Impairment of spatial memory	[68]
	*IP3R2* gene deletion	LTD	Impairment of spatial working memory, fear memory, and recognition memory	[89]
	Expression of dnSNARE in astrocyte	Theta wave synchronization in the hippocampal and prefrontal cortex	Impairment of spatial working memory and recognition memory	[89]
	Light-activated adenylate cyclase increases cAMP levels in astrocytes	LTP	Improvement of spatial memory	[61]
	Overexpression of Piezo1 in astrocyte	LTP	Enhancement of spatial memory	[62]
	Specific deletion of CB1R in hippocampal astrocyte	LTD	Spatial working memory impairment induced by exogenous cannabinoids	[66]
Aversive memory	Chemical or optogenetics activation of Gq GPCR signals in CA1 astrocyte	LTP	Enhancement of contextual fear memory	[116]
	Chemogenetic activation of Gq GPCR signals in basolateral amygdala (BLA) astrocyte	Amygdala prefrontal cortex communication	Enhancement of cued fear memory	[101]
	Optogenetics activation or inhibition of Rac1 activity in basolateral amygdala astrocytes	Inhibiting neuronal activation (reducing neuronal excitability)	Suppressing fear memory acquisition	[118]
	*Chrm1* gene deletion in astrocyte	Hippocampal neurogenesis	Contextual fear memory impairment	[119]
	Specificity Knocking out α7-nAChR of astrocytes in the auditory cortex	Astrocyte calcium signal	Persistent impairment of fear memory	[121]
	Specific knocking out glucocorticoid receptor (GR) in astrocyte	Glucose metabolism	Contextual fear memory impairment	[122]
	Pharmacological inhibition of glycogen decomposition of astrocyte in anterior cingulate cortex (ACC) and short-term activation of astrocyte in ACC	L-lactic acid	Impaired aversion memory related to pain	[123]
	Optogenetics activation of ACC astrocyte β2ARs Chemogenesis activates the Gi pathway in astrocytes and AAV, knocking down astrocyte β2AR	Locus coeruleus (LC) - anterior cingulate cortex ACC neural circuit	Promoting pain-related aversion memory Inhibiting pain-related aversion memory	[124]
Recognition memory	Inhibition of Ca^2+^dependent vesicle release in astrocyte	Gamma oscillation	Impairment of recognition memory	[87]
	The specific knockout of phagocytic receptor MEGF10 in astrocyte	Astrocyte phagocytosis synapse, LTP, LTD	Impairment of recognition memory	[80]
	The specific knockout of astrocyte CX43	Glutamine transfer	Impairment of recognition memory	[125]
	Specific deletion of IGF-IR in cortical astrocyte	LTP, LTD	Impairment of recognition memory	[71]
	Deletion of CB1 receptor in astrocyte	LTP and D-serine	Impairment of recognition memory	[60]
	Knockout of Bmal1 in astrocyte	GABA signaling	Impairment of recognition memory	[127]

## LIST OF ABBREVIATIONS

A1Rs: Adenosine Type 1 Receptor
AD: Alzheimer’s Disease
BDNF: Brain-derived Neurotrophic Factor
CB1R: Cannabinoid Receptor Type 1
Cx30: Connexin 30
GABA: γ-aminobutyric Acid
GPCR: G protein-coupled Receptors
IL-33: Interleukin-33
LTP: Long-term Potentiation
PTSD: Posttraumatic Stress Disorder
STDP: Spike-time Dependent Plasticity
TBS: Theta Burst Stimulation
α7nAChR: α7-nicotine Acetylcholine Receptor
